# Respiratory Burst and TNF-α Receptor Expression of Neutrophils after Sepsis and Severe Injury-Induced Inflammation in Children

**DOI:** 10.3390/ijerph18042187

**Published:** 2021-02-23

**Authors:** Janusz P. Sikora, Jarosław Sobczak, Dariusz Zawadzki, Przemysław Przewratil, Anna Wysocka, Monika Burzyńska

**Affiliations:** 1 Department of Pediatric Emergency Medicine, 2nd Chair of Pediatrics, Central Clinical Hospital, Medical University of Łódź, 36/50 Sporna St., 91-738 Łódź, Poland; jaroslaw.sobczak@umed.lodz.pl (J.S.); dariusz.zawadzki@umed.lodz.pl (D.Z.); 2 Department of Management and Logistics in Healthcare, Medical University of Łódź, 6 Lindleya St., 90-131 Łódź, Poland; 3 Department of Pediatric Surgery and Oncology, Chair of Surgical Pediatrics, Central Clinical Hospital, Medical University of Łódź, 36/50 Sporna St., 91-738 Łódź, Poland; przemyslaw.przewratil@umed.lodz.pl (P.P.); anna.wysocka@umed.lodz.pl (A.W.); 4 Department of Epidemiology and Biostatistics, Chair of Social and Preventive Medicine, Medical University of Łódź, 7/9 Żeligowskiego St., 90-752 Łódź, Poland; monika.burzynska@umed.lodz.pl

**Keywords:** neutrophils, respiratory burst, SIRS, ROS, TNF-α receptor

## Abstract

Systemic inflammatory response syndrome (SIRS) is defined as the systemic host response to infection or a non-infectious factor. The purpose of this study was to evaluate the involvement of reactive oxygen species (ROS) in severe inflammation and to assess the discrimination strength of the neutrophil BURSTTEST assay regarding its etiology in three groups of patients (sepsis, burns, and bone fractures) who met the SIRS criteria. The neutrophil activation (respiratory burst of granulocytes as well as p55 and p75 tumor necrosis factor (TNF-α) receptor expression) was evaluated twice using flow cytometry, and the results were compared with healthy controls and among SIRS subjects. A decreased oxygen metabolism in neutrophils after *E.* *coli* stimulation and increased TNF-α receptor expression were found in septic and burned patients on admission, while ROS production augmented and TNF-α receptor expression diminished with the applied therapy. The significant differences in neutrophil respiratory burst intensity among septic and burned patients and those with sepsis and bone fractures were found (however, there were not any such differences between patients with thermal and mechanical injuries). This study indicates that the neutrophil BURSTTEST evaluation might be a clinically reliable marker for differentiating the SIRS etiology.

## 1. Introduction

Sepsis and severe injuries are known to be common causes of morbidity and mortality in critically ill children. The systemic inflammatory response to both infectious and non-infectious stimuli involves the activation of leukocytes and other inflammatory cells, leading to a massive production of reactive oxygen species (ROS). In the case of an unfavorable and uncontrolled protective systemic reaction of the host, the excessively released endogenic inflammation mediators (e.g., cytokines and ROS) cause various tissue damage and result in the development of multiple organ dysfunction syndrome (MODS). This syndrome is thought to be the most severe complication of systemic inflammatory response syndrome (SIRS), independently of the acted stimulus. A so-called “respiratory burst” of neutrophils, which is a consequence of the activation of leukocytes, dendritic cells, and vascular endothelium, is actually considered the basis of SIRS pathogenesis during sepsis or severe injuries [1,2,3,4,5]. Stimulation of these cells and their interaction is possible due to Toll-like receptors (TLR), which through cells of innate immune response recognize the pathogen-associated molecular patterns (PAMPs) or damage-associated molecular patterns (DAMPs) observed during injuries. The TLR activation through PAMPs and DAMPs, as well as through subsequent transmission of signals capable of inducing defense gene expression, including synthesis-coding proinflammatory cytokines, chemokines, adhesive and co-stimulating molecules, leads to the synthesis of endogenous inflammatory mediators [6,7,8,9].

In a further stage of the host immune response, proinflammatory cytokines (e.g., TNF-α, IL-1, IL-6, IL-8, IL-12, and IL-18) may, among others, stimulate neutrophil respiratory burst. Moreover, it has been recognized that neutrophils participate in subsequent modulation of adaptive immune responses in severe inflammation that can result in immune paralysis through various mechanisms [10,11]. In recent years, activation of neutrophil respiratory burst and mechanisms of microbial killing have been reviewed [12,13]. However, the role of neutrophil activation during sepsis and severe injuries has not been fully explained. There are conflicting reports in the literature concerning this phenomenon [2,3,14,15,16].

This study was proposed with the objective of evaluating the involvement of ROS in SIRS pathogenesis and assessing the discrimination strength of the neutrophil BURSTTEST assay in three groups of pediatric patients (sepsis, burns, and bone fractures) who met the SIRS criteria. In order to assess the discrimination strength of BURSTTEST regarding the etiology of severe inflammation, we decided to research groups of patients with SIRS of infectious (sepsis) and non-infectious origin (burns and bone fractures).

Our study indicates that the neutrophil BURSTTEST evaluation might be considered as a reliable marker for differentiating the SIRS etiology. Moreover, we hypothesized that some clinical cases might be potential candidates for immune-modulating therapy.

## 2. Subjects and Methods

### 2.1. Study Site and Subjects

The study included 46 pediatric patients at different ages with SIRS during the course of sepsis, severe thermal or mechanical injuries (17 septic children, 17 burned children, and 12 children with bone fractures), hospitalized in the Department of Intensive Care and Anesthesiology, and Department of Pediatric Surgery and Oncology of the Central Clinical Hospital, Medical University of Łódź, Poland, from February 2015 to October 2016. Written informed consent was obtained from the children’s parents. The study was conducted in accordance with the Declaration of Helsinki, and the protocol (No. RNN/88/04/KE) was approved by the Medical University of Łódź Ethics Committee. The age of the patients on admission ranged from 3 weeks to 18 years. The control group included 38 age-matched healthy children (newborns (*n* = 17), children with a mean age of 3 years and 1 month (*n* = 11), and children with a mean age of 15 years and 5 months (*n* = 10) (Table 1).

Inclusion criteria included all patients between the ages of 3 weeks and 18 years, and males or females who met the SIRS criteria, according to the International Pediatric Sepsis Consensus Conference in 2002 [17]. Although new adult definitions and criteria were published (Sepsis-3) in 2016, with “sepsis” defined as life-threatening organ dysfunction caused by a dysregulated host response to infection, and application of Sepsis-3 to children has been attempted, formal revisions to the 2005 pediatric sepsis definitions remain pending [18]. Exclusion criteria in our study included immunodeficiencies, malignancies, autoimmune diseases, chronic inflammatory diseases, and lack of the written consent to study. The septic group consisted of 17 neonates (among them 13 were pre-terms), born after 25 to 39 weeks of gestation, with birth weights of 800 to 4000 g and Apgar scores from 1 to 10 points. Sepsis developed after the first week of life and was diagnosed based on clinical symptoms (organ dysfunction, hypoperfusion, hypotension, oliguria, or acute altered mental status) and laboratory test results (e.g., complete blood count (CBC) with a white blood cell (WBC) count manual differential; procalcitonin (PCT); C-reactive protein (CRP); lactic acidosis; and bacteriological results of the blood, urine, stool, cerebrospinal fluid, and discharge from the trachea intubation). Whereas, septic shock was diagnosed in cases of apnea, anuria, paleness of skin surface, metabolic acidosis, capillary refilling time >3 s, and mean blood pressure <20 mmHg; in 4 patients who developed MODS, the disease outcome was fatal. The septic children were treated with wide-spectrum antibiotics (semisynthetic penicillins and aminoglycosides), modified with glycopeptides and cephalosporines in accordance with the results of the bacteriological blood tests and antibiotic resistance of bacterial species. In the cases of septic shock, additional pharmacotherapy was applied, i.e., catecholamines, intravenous immunoglobulins, and pentoxifylline (Table 2).

The burned group consisted of 17 children, aged 24–48 months. The size of the burns accounted for 10–25% of the total body surface area (TBSA) (these were second- and third-degree burns). Fourteen children presented IIa/IIb/III-degree burns, while three patients presented IIa/IIb-degree burns. During the diagnosis, a detailed medical history and physical examination were taken into account. The American Burns Association criteria and the Jackson’s scale were used for size and depth of the burn and rated as severe. They concerned upper and lower limbs, neck, thorax, perinea, feet, and hands. Out of all the burns, seven patients underwent conservative treatment, and ten children underwent epidermal skin grafts after prior excision of necrotic tissues; they were usually performed 5–6 days after the burn (Table 2). Five children experienced hypovolemic shock, although there were no signs of sepsis or MODS in the burned patients.

The group of patients with bone fractures consisted of 12 children, aged 13–18 years; these were the patients with multilocal bone fractures of the upper (5 patients) and lower limbs (5 patients) as well as the facial skeleton (2 patients). Severe mechanical injury was defined as an event that clinically induced SIRS development. Bone fractures were accompanied by soft tissue bruising and hematomas in the course of the generalized injury. Among all the fractures, surgical procedures were performed in 9 children; the remaining injuries were treated by using a conservative method. Surgical treatment was associated with the repositioning of fractured bone fragments and intramarrow stabilization, while the conservative treatment depended on the fractured bone reposition and plaster-of-Paris cast immobilization (a surgical procedure was carried out soon after the injury, considering clinical indications within 24–48 h of treatment). No complications (shock, MODS, or sepsis) appeared in the group of children with bone fractures.

### 2.2. Methods and Collection of Specimens

Blood was collected by venipuncture at two time intervals: the first determination of the immunological markers studied was performed at the time of sepsis diagnosis (prior to the pharmacotherapy), or within the first 6–24 h after thermal or mechanical injury; the second one directly after the completion of treatment (in case of sepsis, 12–24 h after the last antibiotic dose). In the case of a patient’s death, a single assessment of the tested markers was performed, similarly to children from the control group (those single measurements of the tested parameters in the deceased patients were included in the statistical analysis—Table 3). The average interval of the second measurement following SIRS equaled 14 days after sepsis, 11 days after burn injury, and 9 days after bone fractures, and was a consequence of data obtained from the clinical trial (duration of SIRS and its resolution) and the normalization of the CRP levels.

First, the heparinized whole blood samples (1.0 mL) were collected from our patients, followed immediately by the neutrophil respiratory burst assay (BURSTTEST) along with the expression of the TNF-α receptor on these cell surfaces. The analysis of the immunological markers studied using flow cytometry was performed in the Department of Clinical Immunology at the Medical University of Łódź.

#### 2.2.1. Neutrophil Respiratory Burst Assay (BURSTTEST)

Flow cytometry was used to evaluate the neutrophil respiratory burst with a BURSTTEST reagent kit (ORPEGEN Pharma, Heidelberg, Germany). A FACSCalibur cytometer with argon laser at a wavelength of 488 nm (Becton Dickinson, CA, USA) was applied in order to examine the ROS production. The CELLQuest programme (Becton Dickinson) was used to analyze the achieved results that were shown as the median fluorescence intensity (MFI) of phorbol 12-myristate 13-acetate (PMA) or *E. coli*-stimulated neutrophils.

A quantitative determination of the neutrophil oxidative burst was possible due to the BURSTTEST; dihydrorhodamine (DHR) 123 served as a fluorogenic substrate while unlabeled opsonized *E. coli* bacteria were used as the particulate stimulus. PMA or *E. coli* was incubated with heparinized whole blood at 37 °C and as a negative background control a sample without stimulus was used. ROS (i.a., hydrogen peroxide, hypochlorous acid, and superoxide anion) were produced by neutrophils upon stimulation and they were responsible for intraphagosomal bacterial killing; this phenomenon takes place in the mitochondria as DHR 123 can be oxidized by H_2_O_2_ and O_2_^−^ to rhodamine 123, and then monitored following the reactive oxidants synthesis during respiratory burst in the neutrophils. Next, a bright fluorescent signal upon excitation by blue light (488 nm) was emitted as a consequence of the biochemical reaction described above. It was stopped by the addition of LYSING SOLUTION, which removes erythrocytes and contributes to a partial neutrophil fixation. DNA STAINING SOLUTION is added to exclude aggregation artifacts of bacteria or cells after one washing step with WASHING SOLUTION. The MFI following the interaction of reactive oxygen radicals is then measured.

The BURSTTEST assay procedure has been thoroughly described by the authors of this paper in their earlier report [19].

#### 2.2.2. The Neutrophil p55 and p75 TNF-α Receptor Expression Assays

Fluorescein (Fluorochrome-anti-human TNFR I monoclonal antibody, R&D, Minneapolis, USA) and phycoerythrin (Fluorochrome-anti-human TNFR II monoclonal antibody, R & D, Minneapolis, USA) conjugated with monoclonal antibodies were used to evaluate the neutrophil p55 and p75 TNF-α receptor expression. The vacuum tubes were filled with heparinized whole blood, and serum contamination was then removed by washing the cells three times with isotonic phosphate buffer by centrifugation at 500 *g* for 5 min. After transferring 50 µL of the packed cells to a 5 mL tube for staining with the monoclonals, following their prior Fc-blocking by treatment with 1 µg of human IgG/10^5^ cells, they were placed for 15 min at room temperature. Then, a 5 mL tube was filled with 25 µL of the Fc-blocked cells (1 × 10^5^ cells). Finally, 10 µL of fluorescein-conjugated anti-TNFR I or phycoerythrin-conjugated anti-TNFR II reagent was added to allow the tubes’ incubation for 30–45 min at 2–8.0 °C. Unreacted anti-TNFR I or anti-TNFR II reagent was removed by washing the cells twice in 4 mL of the PBS buffer (whole blood required a RBC lysis step, using a lysing reagent (R&D System Whole Blood Lysing Kit)), following the incubation procedure. The cells were then resuspended in 200–400 µL of PBS buffer in order to evaluate the neutrophil p55 and p75 TNF-α receptor expression on their surfaces by means of flow cytometry with 488 nm wavelength laser excitation. The CELLQuest programme was applied to analyze the achieved data; the results were expressed as neutrophil MFI in relation to the cell population with expressed p55 and p75 receptors.

### 2.3. Statistical Analysis

The research results were described as the MFI of PMA or *E. coli*-stimulated neutrophils, presenting the intensity of the neutrophil oxygen burst, while the MFI of cells expressing p55 and p75 receptors showed activation of neutrophils following their stimulation. A Shapiro–Wilk test revealed that the distribution of the data of the variables measured was not normal; thus, non-parametric tests were used in our further statistical analysis. Wilcoxon’s test was applied in the cases of dependent variables (data were compared in groups of patients that met the SIRS criteria in the dynamics, i.e., the first determination of the studied immunological markers at the time of sepsis diagnosis, or shortly after thermal or mechanical injury, and the second one following the completion of treatment). The independent variables were analyzed with Mann–Whitney’s test (sick children’s data were compared to data of children from the control group) or with a nonparametric equivalent of the one-way analysis of variance (ANOVA test, Kruskal–Wallis rank) that was applied to the comparative analysis of the neutrophil fluorescence intensity in patients with sepsis, burns, or bone fractures, as well as to compare the variables in the control groups. Multiple mean rank comparison tests for all samples were used as post-hoc tests. 

The differences were considered significant at *p* < 0.05. The statistical analysis was made with STATISTICA 10.0 PL (StatSoft, Inc., Tulsa, OK, USA).

## 3. Results

### 3.1. Respiratory Burst of Neutrophils in SIRS Patients and in Controls

**Figure 1:**
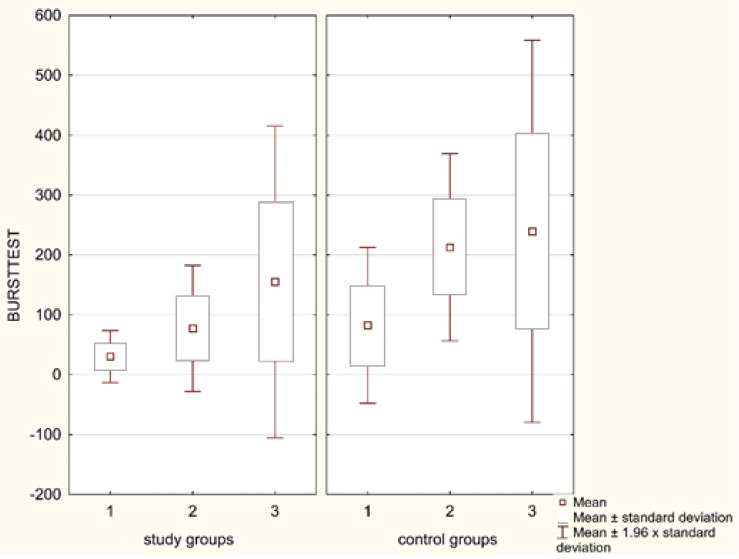
Neutrophil respiratory burst after *E.coli* stimulation (in the studied children with sepsis (Group 1), burns (Group 2), and bone fractures (Group 3) at the time of diagnosis and in the control groups).

Statistically significant differences were revealed in the neutrophil respiratory burst intensity after *E. coli* stimulation assessed in 3 groups of patients, both prior to treatment (within 6–24 h) (*p* = 0.0029) as well as after its completion and CRP normalization (*p* = 0.0001). The patients with sepsis presented, both prior to and after the treatment, significantly lower MFI values for *E. coli*-stimulated granulocytes as compared to the burned patients (*p* = 0.0013, *p* = 0.0001, respectively) (Table 3 and Table 4). Similarly, the burst intensity in children with sepsis prior to and after the treatment was significantly lower than in children with bone fractures (*p* = 0.0089, *p* = 0.0008, respectively). However, the comparison of the neutrophil oxygen metabolism between the burned children and those with bone fractures did not reveal any statistically significant differences in the time periods analyzed (*p* = 0.6927 prior to and *p* = 0.4431 after the treatment). Thus, the lowest production of ROS during neutrophil respiratory burst was observed in the course of SIRS induced by an infectious factor (sepsis), whereas the patients with bone fractures presented the least reduction in neutrophil oxygen metabolism (Figure 1).

**Figure 2:**
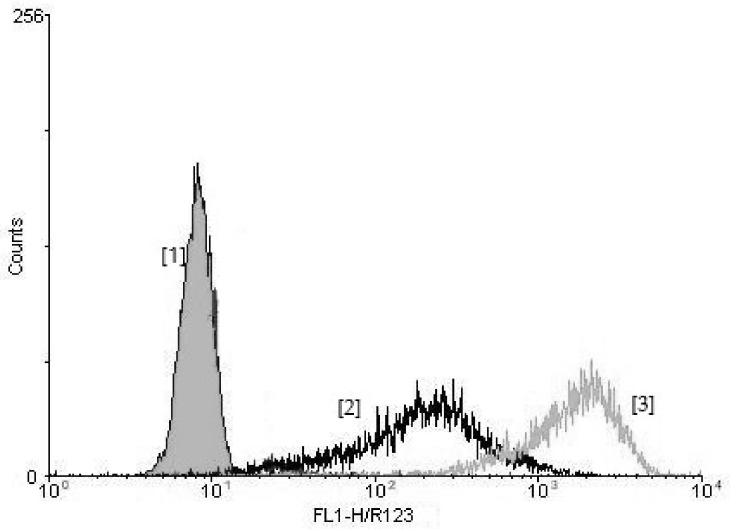
The rhodamine 123-fluorescence intensity of neutrophils without stimulation (control) [1] and exposed to *Escherichia coli* [2] and PMA [3] (in relative fluorescence units, i.e., the median channel of the fluorescing cell population).

Flow cytometry was used to analyze the rhodamine 123-fluorescence and a logarithmic scale was applied in order to display the fluorescence features of the cells studied (Figure 2). To include neutrophils and exclude most of the mononuclear cells (identified on the basis of the light scattering properties of the cells), the same numbers (10^4^ events) were taken into consideration by means of a gate drawn during each flow cytometric analysis.

**Figure 3:**
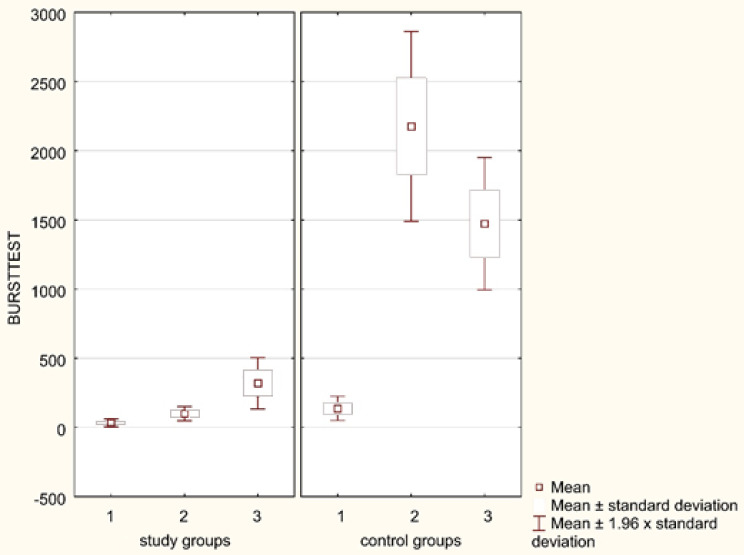
Neutrophil respiratory burst after PMA stimulation (in the studied children with sepsis (Group 1), burns (Group 2), and bone fractures (Group 3) at the time of diagnosis and in the control groups).

While examining the respiratory burst of neutrophils after their stimulation with PMA, the same relations were observed in three comparable groups within the analogical time periods. Prior to and after the treatment, the neutrophil MFI following PMA stimulation was significantly (*p* = 0.0291, *p* = 0.0025, respectively) lower in patients with sepsis as compared to burned children (Figure 3). A similar phenomenon was noticed while comparing neutrophil oxygen metabolism in septic children with those with bone fractures in the time periods analyzed (*p* = 0.0011, *p* = 0.0005, respectively). However, there were no statistically significant differences regarding the neutrophil burst intensity (both prior to and after the treatment) between the patients with burns and those with bone fractures (Table 3 and Table 4).

A significant increase in the neutrophil respiratory burst (*p* < 0.05) after the therapy termination as compared to the initial values (MFI in *E. coli*-stimulated neutrophils were 34.2 vs. 63.3 and 70.4 vs. 235.0, respectively) was observed both in the septic and burned children. The oxygen metabolism of neutrophils after PMA stimulation in children with bone fractures significantly (*p* < 0.05) rose with the applied therapy (MFI was 333.1 vs. 2571.4); after stimulation of neutrophils with *E. coli*, the results were not statistically significant (Table 3 and Table 4).

Significantly (*p* < 0.001) lower values of MFI after *E. coli* stimulation were observed, both in septic and burned children (within the first 6-24 h after sepsis or burn diagnosis) in comparison with the control groups (MFI was 34.2 vs. 77.3 and 70.4 vs. 228.8, respectively). As to the consequences of neutrophil activation in patients with bone fractures (within the first 6–24 h after injury) and in the controls, no statistically significant values of neutrophil MFI after *E. coli* stimulation were revealed. However, after the cell stimulation with PMA, the initial neutrophil respiratory burst intensity in that group of patients was significantly (*p* < 0.05) lowered in comparison with the controls (MFI was 333.1 vs. 1584.9) (Table 3, Table 4 and Table 5).

As to the neutrophil activation in the control groups, 38 healthy children constituted an additional three control groups differentiated with regard to age. A statistically significant correlation for the age and the BURSTTEST intensity after *E. coli* (*p* = 0.0016) and PMA (*p* = 0.0292) neutrophil stimulation was observed (Table 5).

### 3.2. TNF-α Receptor Expression of Neutrophils in SIRS Patients and in Controls

The analysis of the p55 and p75 TNF-α receptor expression did not reveal statistically significant differences prior to the treatment in the three groups studied (Table 3). However, statistically significant differences were seen (*p* = 0.0000 for p55, and *p* = 0.0083 for p75) while comparing the TNF-α receptor expression after the treatment and CRP normalization in the same patients. The p55 expression was significantly lower in children with sepsis comparing to those with burns (*p* = 0.0000) and to the patients with bone fractures (*p* = 0.0064); there were also statistically significant differences between the burned patients and those with bone fractures (*p* = 0.0024). The assessment of p75 expression after the treatment completion resulted in significant differences between the group of children with sepsis and the burned children (*p* = 0.0004); no statistically significant differences were found between the septic patients and those with bone fractures as well as the burned children and those with mechanical injuries (Table 4).

The neutrophil expressing p55 and p75 TNF-α receptors were cytometrically examined by means of fluorescein-conjugated anti-TNFR I or phycoerythrin-conjugated anti-TNFR II reagent as the fluorescence source. The intensity of fluorescence was proportional to the quantity of fluorescein (FITC)-conjugated anti-TNFR I or phycoerythrin (PE)-conjugated anti-TNFR II monoclonals that they have been attached to. The cells expressing p55 and p75 receptors demonstrated a strong specific fluorescence, characteristic of the corresponding fluorochrome, while a group of cells without expression of p55 and p75 receptors served as the control.

The expression of TNF-α receptors on the neutrophils significantly (*p* < 0.05) diminished, both in septic patients (MFI of cells expressing p55 and p75 receptors was 15.42 vs. 3.94 and 30.53 vs. 8.31, respectively) and burned children (MFI of cells expressing p55 receptor was 13.95 vs. 11.44; the MFI values for p75 did not differ), taking into account the groups of subjects studied and the corresponding duration of observation. Furthermore, the p55 receptor expression significantly (*p* < 0.05) decreased after the treatment in that group of patients (MFI was 14.86 vs. 10.6), while p75 receptor expression did not differ (Table 3 and Table 4). On the contrary, the initial expression of TNF-α receptors on neutrophils was significantly (*p* < 0.05) augmented in septic patients (MFI of cells with p55 and p75 receptor expression was 15.42 vs. 9.41 and 30.53 vs. 13.12, respectively) as compared to controls. While, in burned patients, no statistical significance for p55 and p75 receptor expression between the compared groups was found (Table 3 and Table 5). Assessing neutrophil activation in patients with bone fractures (within the first 6–24 h after injury) and in the controls, no statistically significant values of the MFI of cells with p55 and p75 receptor expression were observed.

Considering the activation of neutrophils in the controls, it was revealed that the receptor expression for TNF-α on these cells significantly (*p* = 0.0033 for p55; *p* = 0.0001 for p75) changed with age in the three control groups: newborns (*n* = 17), children with a mean age of 3 years and 1 month (*n* = 11), and children with a mean age of 15 years and 5 months (*n* = 10) (Table 5).

## 4. Discussion

Contemporary studies concerning SIRS demonstrate the complexity of the pathophysiological mechanisms accompanying this syndrome. A systemic inflammatory response is defined by immune cell activation and elevated levels of proinflammatory cytokines, which are to defend the organism; however, if they are generated in excess, they may become harmful. Therefore, induction of a compensatory anti-inflammatory response (CARS) will affect homeostatic processes and is characterized by increased levels of anti-inflammatory cytokines and immunoparesis [10]. Production of anti-inflammatory cytokines and cytokine inhibitors seems thus to be an initial condition to control and limit the defense response of patients with a systemic inflammatory process. Whereas, disorders in the ROS generation and inactivation at the time of the inefficient antioxidative barrier lead to the development of the so-called “oxidative stress”. In consequence, this results in tissue and organ impairment, and clinically the development of syndromes such as disseminated intravascular coagulation (DIC), adult respiratory distress syndrome (ARDS), or MODS [20]. It is worth mentioning that the following factors play an important role in SIRS pathogenesis: microorganism virulence, injury extensiveness, comorbidities in the patient, or gene polymorphism responsible for production of cytokines and other effectors of the immune response [10,21,22].

In our patients, we evaluated the activation of peripheral blood neutrophils, i.e., changes in the neutrophil oxygen metabolism and the TNF-α receptor expression on those cells. Literature reports concerning this subject are often conflicting [2,3,14,15,16]. Some authors observed increased neutrophil and monocyte oxygen metabolisms in septic patients, which changed according to the stage of the disease [14,15]. Santos et al. evaluated ROS generation by neutrophils and correlated their levels with clinical outcomes, revealing that neutrophils from adult patients (the mean age was 60 years) with fatal sepsis were characterized by markedly increased production of ROS, and its persistence was associated with a poor outcome [15]. However, other studies reported that a decreased ROS production by neutrophils may be observed in very severe cases of sepsis, frequently with the occurrence of a septic shock [3,23]. By assessing the activity of neutrophil oxygen metabolism in various pathological conditions, Drossou et al. showed that prematurity, sepsis, and stress significantly reduce the intensity of the respiratory burst of granulocytes [23]. These findings are in line with our research in which we observed a lower ROS production in neonates at the time of sepsis diagnosis, and a significant increase in the ability of those cells to initiate the respiratory burst after the treatment completion. The conducted individual analysis also showed that the four neonates with sepsis who did not survive (developed septic shock and MODS in the further course of the disease—Table 2) presented an extremely low respiratory burst of neutrophils—the MFI value after stimulation of *E. coli* in these patients was 6.9 (*p* = 0.0415, Mann–Whitney test). Statistical analysis using the same test showed significantly lower MFI values of neutrophils after stimulation with *E. coli* at diagnosis of sepsis compared to the control group (34.2 vs. 77.3; *p* < 0.0001) (Table 3 and Table 5). Similarly, significantly lower oxygen metabolism of granulocytes was observed in patients after the end of antibiotic therapy as compared to the control group (63.3 vs. 77.3; *p* = 0.018). Additionally, in our research we indicate that the evaluated p55 and p75 receptor expression on neutrophils was significantly lowered during the treatment and differed from that in the control group, which may be a subsequent confirmation of the TNF-α involvement in sepsis pathogenesis (Table 4 and Table 5).

In the complex pathogenesis of trauma, more and more attention has been drawn towards the action of proinflammatory cytokines, released by activated phagocytes, and ROS produced as a result of oxidative stress [2,16,24,25,26]. Recently, elevated levels of pro- and anti-inflammatory cytokines have been measured in samples obtained 2–24 h post-injury, and the investigators have questioned whether patients with a more robust inflammatory response to trauma are at an increased risk of adverse outcome [24,25]. Due to some difficulties in the reconstruction of the ROS presence and activity in vivo (as they have a very short half-life), ROS involvement in the SIRS pathomechanism in patients after trauma is still not well documented. Literature reports on this subject are not complete or univocal. Two decades ago, Rosenthal et al. observed a reduced neutrophil respiratory burst while examining the cytosolic components (p47-phox and p67-phox) of NADPH:O_2_ oxidase in patients with thermal injury [27]. Whereas, Liao et al. showed increased free radical production in leukocyte homogenates and elevated expression of NADPH oxidase (gp91(phox)) in circulating leukocytes, indicating an intense induction of oxidative burst following traumatic brain injury that may lead to systemic damage [2]. On the other hand, Hietbrink et al. investigated blood samples of injured patients that were analyzed for activation of neutrophils and showed a gradual decrease in expression of fMLF-induced active FcγRII before clinical signs of septic shock [16]. Our own studies indicated significantly higher neutrophil ability to initiate the respiratory burst after the treatment completion as compared to the baseline (low ROS production) just after burn injury—the MFI values of the neutrophils after *E. coli* stimulation were 235.0 vs. 70.4 (Table 3 and Table 4). This reduced neutrophil oxygen metabolism on SIRS diagnosis can be explained by a release of a large amount (similarly to sepsis) of endogenous inflammatory mediators (proinflammatory cytokines, LTB_4_, and PAF), and due to their activity by running out granulocyte functional reserves during the course of extensive thermal injury. Moreover, uncontrolled ROS release by neutrophils in an inflammatory microenvironment can lead to parallel tissue damage by excessive degranulation and proteases release [4]. Boomer et al. revealed that patients who died in the intensive care unit following sepsis compared with those with non-sepsis etiologies have biochemical, flow cytometric, and immunohistochemical findings consistent with immunosuppression [11].

Diminished receptor expression for TNF-α (p55 and p75), observed by us after sepsis treatment, as well as for p55 following burn or mechanical injury therapy (Table 4), may confirm TNF-α involvement in the pathomechanism of SIRS development initiated by infectious or non-infectious factors. Recently, several studies have shown elevated levels of IL-6, IL-8, IL-10, granulocyte colony-stimulating factor (G-CSF), and IL-1 receptor antagonist (IL-1 RA) in serum samples of non-survivors compared to survivors while measuring the concentrations of cytokines in the early post-burn injury phase [24,28]. These data indicate the prognostic value of the examined indices. On the other hand, Santos et al. indicated elevated ROS production by neutrophils of septic patients to be correlated with clinical outcomes [15].

In our study, however, no significant differences between the p55 and p75 TNF-α receptor expression on neutrophils in children with thermal and mechanical injuries were observed as compared to the control group, which may demonstrate a less significant TNF-α involvement in these patients’ granulocyte activation as opposed to, e.g., children with sepsis (for whom statistically significant differences in receptor expressions as compared with the controls were recorded). Likewise, the investigation of the neutrophil respiratory burst after *E. coli* stimulation in patients with bone fractures did not reveal any statistically significant differences in the MFI of these cells observed at the time periods analyzed, nor in comparison with the control group (Table 3 and Table 4). Whereas, after the neutrophil stimulation with PMA in this group of patients, statistically significant differences were noted (MFI values increased along with the treatment applied and were lower in children soon after the injury as compared to the controls) (Table 3, Table 4 and Table 5). This observation demonstrates that stimulation of neutrophils via the extrareceptor route plays a crucial role in activation of these cells in the course of SIRS after bone fractures. However, literature reports on ROS involvement in the development of systemic inflammatory response in injuries are still rare and ambiguous [2,16,27].

Why do people react to injury or other inflammatory and infectious stimuli in a different way? Why do some patients have severe clinical signs of a systemic inflammatory reaction leading to death, and the others activate local defense mechanisms limiting the inflammatory response, thus avoiding the development of generalized inflammation? These problems are the subject of intensive research all over the world.

In a new approach to the SIRS concept, one of the main factors determining the development of the systemic inflammatory reaction and its further course is, among others, an individual susceptibility to develop sepsis affected by gene polymorphism, concerning, e.g., Toll-like receptors, cytokines (TNF-α, TNF-β, IL-1), or clotting factors [21,29,30,31,32]. These studies are aimed at revealing a relationship between a genotype determined by the examined gene polymorphism and severity and the further course of SIRS. The continuation of these studies is supposed to provide further inspiration for creating new therapeutical strategies and will lead to individualization of SIRS treatment in particular patients. To achieve this goal, it is necessary to specify the SIRS etiological factor and find adequate markers that would enable us to differentiate this factor. Considering the abovementioned postulate, it should be added that the age of the patient is also important. Both the youngest children (especially premature newborns) and the elderly are at risk of developing generalized septic conditions due to immunosuppression. In these groups of patients, very often the local inflammatory reaction is ineffective and generates the clinical symptoms of SIRS. It should also be remembered that the result of minor injuries is a local defensive reaction of the organism, while extensive injuries lead to a general response.

Our study partially meets the presented expectations. Statistically significant differences concerning MFI values of the neutrophil respiratory burst in three groups of pediatric patients indicate the possibility of differentiating the neutrophil oxygen metabolism intensity during SIRS (in the course of sepsis, severe burns, or bone fractures). We noticed the diminished oxygen metabolism of neutrophils both in the septic children and the severely injured patients, although the lowest statistically significant MFI values of neutrophil respiratory burst were observed in the course of SIRS induced by an infectious factor (sepsis)—the MFI of *E. coli-*stimulated neutrophils was 34.2 (Table 3). Patients with SIRS who presented with remarkably low oxidative metabolism upon diagnosis (low BURSTTEST values), e.g., children with sepsis, are potential candidates for immune-modulating therapy. Assuming that the diminished ROS production in these patients is due to functional neutrophil exhaustion, as a consequence of multiple cell stimulation by proinflammatory cytokines produced in large quantities, their excess production should be inhibited. In order to do so, pentoxifylline, which is a TNF-α inhibitor, treatment may be applied. Other methods of restoring neutrophil energetic potentials and increasing their ability to produce ROS may be attempted by using granulocyte colony-stimulating factor (G-CSF), which stimulates not only its quantity but also improves cell function (including bactericidal abilities dependent on oxidative mechanisms of destruction). Taking into account the fact that steroids impede proinflammatory cytokine production, their application should be considered in chosen cases (e.g., septic shock resistant to fluids and catecholamines). Thus, targeted immune-modulating therapy may be a valid approach in selected patients with severe inflammation.

## 5. Conclusions

Our studies confirm the involvement of ROS in SIRS pathogenesis in the course of sepsis and severe thermal and mechanical injuries. Therefore, we suggest that the evaluation of neutrophil oxygen metabolism in different clinical entities, as a supplement to the anamnestic data and imaging studies, creates the possibility of differentiating the SIRS etiology.

## Figures and Tables

**Table 1:** The characteristics of the studied groups.

Characteristic		Study Groups (*n* = 46)			Control Groups (*n* = 38)		
		Group 1 (*n* = 17)	Group 2 (*n* = 17)	Group 3 (*n* = 12)	Group 1 (*n* = 17)	Group 2 (*n* = 11)	Group 3 (*n* = 10)
Age (Group 1—days; Group 2—months; Group 3—years)	Minimum	21.0	24.0	13.0	21.0	24.0	13.5
	Maximum	28.0	48.0	18.0	28.0	46.0	18.0
	Mean	24.1	34.2	15.5	25.2	37.0	15.4
	Standard deviation	2.1	7.3	1.7	2.3	7.9	1.4
Sex N (fractions)	Male	10 (0.59)	9 (0.53)	8 (0.67)	9 (0.53)	6 (0.55)	7 (0.70)
	Female	7 (0.41)	8 (0.47)	4 (0.33)	8 (0.47)	5 (0.45)	3 (0.30)

**Table 2:** The characteristics of systemic inflammatory response syndrome (SIRS) patients (septic/burned/injured children).

Patient Groups	Sepsis Etiology/ Depth of Burns/ Bone Fracture	Pathogen/ TBSA/ Type of Injury	Treatment	Intervals between Measurements	Complications
Septic neonates (*n* = 17) Burned toddlers (*n* = 17) Injured children (*n* = 12)	Gram-positive bacteria –10 pts Gram-negative bacteria – 7 pts IIa/IIb – 3 pts IIa/IIb/III – 14 pts Upper limbs – 5 pts Lower limbs – 5 pts Facial skeleton – 2 pts	Staphylococcus Klebsiella Pseudomonas Enterobacter Escherichia 10-11 % – 5 pts 15 % – 6 pts 20-25% – 6 pts Multifocal Multifocal Multifocal	Antibiotic therapy – 17 pts Catecholamines, I.V. IG and PTX – 4 pts Necrectomy with dermo-epidermal graft – 10 pts Conservative –7 pts Surgical – 9 pts Conservative – 3 pts	14 days (min. 10 – max.16) 11 days (min. 6 – max. 13) 10 days (min. 5 – max.12)	Septic shock and MODS – 4 pts Hypovolemic shock – 5 pts No complications

Note: pts, patients. PTX, pentoxifylline. I.V. IG, intravenous immunoglobulins.

**Table 3:** Fluorescence intensity of the neutrophils (at the time of sepsis diagnosis or within the first 6–24 h after injury diagnosis) in the studied groups of children with SIRS.

MFI (IQR)	Sepsis (Group 1) (*n* = 17)	Burns (Group 2) (*n* = 17)	Bone Fractures (Group 3) (*n* = 12)	Statistical Analysis ANOVA
MFI of neutrophils after PMA stimulation after *E. coli* stimulation	32.5 (29.4) 34.2 (32.8)	90.6 (151.7) 70.4 (54.9)	333.1 (1281.9) 134.5 (352.3)	*p* = 0.0006 *p* = 0.0291 gr.1 vs gr.2 *p* = 0.0011 gr.1 vs gr.3 NS gr.2 vs gr.3 *p* = 0.0029 *p* = 0.0013 gr.1 vs gr.2 *p* = 0.0089 gr.1 vs gr.3 NS gr.2 vs gr.3
MFI of neutrophils with p55 expression	15.42 (7.10)	13.95 (10.1)	14.86 (16.5)	NS
MFI of neutrophils with p75 expression	30.53 (44.8)	21.67 (8.7)	20.06 (57.1)	NS

MFI, median fluorescence intensity. IQR, interquartile range. vs., versus. gr., group.

**Table 4:** Fluorescence intensity of the neutrophils (after treatment and CRP normalization) in the studied groups of children with SIRS.

MFI (IQR)	Sepsis (Group 1) (*n* = 17)	Burns (Group 2) (*n* = 17)	Bone Fractures (Group 3) (*n* = 12)	Statistical Analysis ANOVA
MFI of neutrophils after PMA stimulation after *E. coli* stimulation	358.1 (165.2) 63.3 (41.3)	2090.8 (858.9) 235.0 (337.6)	2571.4 (1668.8) 366.8 (306.0)	*p* = 0.0003 *p* = 0.0025 gr.1 vs gr.2 *p* = 0.0005 gr.1 vs gr.3 NS gr.2 vs gr.3 *p* = 0.0001 *p* = 0.0001 gr.1 vs gr.2 *p* = 0.0008 gr.1 vs gr.3 NS gr.2 vs gr.3
MFI of neutrophils with p55 expression	3.94 (4.2)	11.44 (4.5)	10.60 (5.9)	*p* = 0.0000 *p* = 0.0000 gr.1 vs gr.2 *p* = 0.0064 gr.1 vs gr.3 *p* = 0.0024 gr.2 vs gr.3
MFI of neutrophils with p75 expression	8.31 (12.4)	20.26 (19.4)	19.46 (10.1)	*p* = 0.0083 *p* = 0.0004 gr.1 vs gr.2 NS gr.1 vs gr.3 NS gr.2 vs gr.3

MFI, median fluorescence intensity. IQR, interquartile range. vs., versus. gr., group.

**Table 5:** The fluorescence intensity of neutrophils in the control groups.

MFI (IQR)	Newborns (*n* = 17)	Children Aged (×) 3 Years and 1 Month (*n* = 11)	Children Aged (×) 15 Years and 5 Months (*n* = 10)	Statistical Analysis ANOVA
MFI of neutrophils after PMA stimulation after *E. coli* stimulation	148.6 (105.4) 77.3 (52.3)	2206.7 (1452.4) 228.8 (144.0)	1584.9 (888.1) 220.2 (271.7)	*p* = 0.0292 *p* = 0.0016
MFI of neutrophils with p55 expression	9.41 (2.3)	12.63 (4.4)	13.28 (4.6)	*p* = 0.0033
MFI of neutrophils with p75 expression	13.12 (3.9)	16.4 (8.7)	18.11 (8.4)	*p* = 0.0001

MFI, median fluorescence intensity. IQR, interquartile range. (×), arithmetic mean.

## Data Availability

The data presented in this study are available on request from the corresponding author. The data are not publicly available.

## Abbreviations

p55 TNF-α receptor	p55 receptor for TNF-α
p75 TNF-α receptor	p75 receptor for TNF-α
PMA	phorbol 12-myristate 13-acetate
CRP	C reactive protein
